# Fear of COVID-19 and religious coping mediate the associations between religiosity and distress among older adults

**DOI:** 10.34172/hpp.2021.40

**Published:** 2021-08-18

**Authors:** Karim Asgari Ghoncheh, Chieh-hsiu Liu, Chung-Ying Lin, Mohsen Saffari, Mark D. Griffiths, Amir H Pakpour

**Affiliations:** ^1^Social Determinants of Health Research Center, Research Institute for Prevention of Non-Communicable Diseases, Qazvin University of Medical Sciences, Qazvin, Iran; ^2^Department of Islamic Studies, Qazvin University of Medical Sciences, Qazvin, Iran; ^3^Department of Geriatrics and Gerontology, Research Center of Clinical Medicine, National Cheng Kung University Hospital, Tainan, Taiwan; ^4^Institute of Allied Health Sciences and Departments of Occupational Therapy and Public Health, National Cheng Kung University Hospital, College of Medicine, National Cheng Kung University, Tainan, Taiwan; ^5^Health Research Center, Life Style Institute, Baqiyatallah University of Medical Sciences, Tehran, Iran; ^6^International Gaming Research Unit, Psychology Department, Nottingham Trent University, Nottingham, UK; ^7^Department of Nursing, School of Health and Welfare, Jönköping University, Jönköping, Sweden

**Keywords:** Anxiety, Coping behavior, Coronavirus infection depressive disorder, Older adults, Religious belief

## Abstract

**Background:** A mediation model was proposed to explain how religiosity, religious coping, and fear of coronavirus disease 2019 (COVID-19) explained anxiety and depression among older adults.

**Methods:** With the use of a cross-sectional design, the Integrated Health System was used to randomly invite 1000 older adults residing in Qazvin to participate in an online survey. Within the period of November 2020 to January 2021, 696 older Iranian adults (mean age=69.56years; 57.9% women) agreed to participate in the study and reported demographic information as well as measures of religiosity, fear of COVID-19, religious coping, anxiety, and depression.

**Results:** Religiosity had direct effects on depression (B [SE]=-0.087 [0.037]; P=0.023) but not anxiety (B [SE]=-0.063 [0.036]; P=0.072). Moreover, both fear of COVID-19 and religious coping significantly mediated the association between religiosity and anxiety (B [SE]=-0.360[0.035]; p=0.002) and that between religiosity and depression (B [SE]=-0.365 [0.034];P=0.002).

**Conclusion:** During the tough time of COVID-19 pandemic, religiosity and religious coping were protectors for older adults in developing good mental. Therefore, future research is needed to examine education programs that are effective for older adults to obtain correct knowledge concerning COVID-19, including the protective COVID-19 infection behaviors. Therefore, older adults may reduce their fear via their enhanced correct knowledge concerning COVID-19.

## Introduction

Older adults have been a vulnerable population during the coronavirus disease 2019 (COVID-19) pandemic. More specifically, this population has higher chances of developing different types of chronic diseases such as hypertension, diabetes, and stroke.^1^ Recent COVID-19 evidence indicates relatively high mortality rates due to COVID-19 infection (8.0%-12.8%) among this population.^2^ Therefore, older adults are likely to report stronger levels of fear during the COVID-19 outbreak period and subsequently develop serious mental health problems if their fear is not carefully taken care of.^3-5^ Additionally, the latest evidence shows loneliness resulting from COVID-19-related virus inhibition policies is a risk factor among older adults in developing anxiety and depression.^6,7^ Moreover, the negative association between loneliness and anxiety/depression are exacerbated when older adults perceive themselves as older.^7^ In this regard, healthcare providers would obtain information regarding the protecting factors for older adults to maintain their mental health during such a tough period.

In order to capture the protectors effectively and efficiently for older adults to maintain their mental health during such a tough period, using a well-developed theory is one of the best solutions.^8^ Therefore, Terror management theory (TMT)^9,10^ was used to design appropriate protectors for older adults’ mental health in the present study. TMT proposes that individuals may use unconscious psychological mechanisms to prevent negative thoughts or feelings and subsequently protect them from psychological distress, such as anxiety and depression.^8,11-13^ Using TMT, religiosity and religious coping were considered to be potentially protect older adults from developing anxiety and depression during the COVID-19 pandemic.

Religiosity, defined as “a person’s behavior and attitudes in relation to a specific religiosity and its rules, values, and practices”,^14^ can be classified into two types: intrinsic religiosity (i.e., the affinity between the religious individuals and their values/beliefs, resulting in a strong bond between the individuals with the embraced creed)^15,16^ and extrinsic religiosity (i.e., the use of religiosity to improve individuals’ health needs with a focus on oneself, such as personal comfort and social relationships).^15,17^ Prior evidence has demonstrated strong associations between religiosity and health (especially mental health) across different populations, including older adults and those with chronic diseases.^14,18-21^

The associations between religiosity and mental health have been proposed to be mediated via religious coping; that is, using beliefs, attitudes, or practices that are based on religiosity to reduce emotional stress through an alternative so that the suffering is meaningful.^22-24^ With the use of religious coping, the suffering becomes bearable.^25^ Indeed, the literature has shown that using positive religious coping, individuals can reduce their psychological distress (e.g., anxiety and depression) and improve their psychological well-being.^19,26^ Moreover, good religious coping may increase individuals’ healthy behaviors (e.g., medication adherence) and quality of life.^20^ Therefore, religious coping could mediate the association between mental health and religiosity.

Aside from religious coping mediated the association between mental health and religiosity, the present study proposes that fear of COVID-19 could be another potential mediator in this association. More specifically, since the beginning of the COVID-19 outbreak, a substantial amount of research has found that individuals worldwide suffer from a variety of psychological distress and fear of COVID-19.^3,27,28^ Moreover, the association between different types of psychological distress such as anxiety and depression and fear of COVID-19 has been verified.^29,30^ Given that religiosity provides individuals with beliefs and religious coping assists individuals in using religiosity to cope with suffering, it is reasonable to postulate that religiosity and religious coping may help individuals reduce their fear of COVID-19. Consequently, fear of COVID-19 appears to mediate the association between religiosity, religious coping, and psychological distress.

In order to assist older adults in maintaining their mental health during COVID-19 pandemic, the present paper proposes a psychological mechanism to explain the psychological distress according to the aforementioned empirical literature concerning religiosity, religious coping, and fear of COVID-19 among older adults during this tough period. In this psychological mechanism, religiosity may have direct and indirect effects on the diminished psychological distress via religious coping and fear of COVID-19. If the evidence supports this psychological mechanism, healthcare providers will have clear information regarding whether it is appropriate to use religiosity and religious coping to help older adults cope with fear of COVID-19 and psychological distress during the COVID-19 pandemic. Based on this psychological mechanism, the following specific hypotheses were formulated: H_1_, religious coping and fear of COVID-19 will be significant mediators in the association between religiosity and anxiety. H_2_, religious coping and fear of COVID-19 will be significant mediators in the association between religiosity and depression.

## Materials and Methods

### Participants and procedure

Older adults living in a city near to Tehran (i.e., Qazvin) were surveyed between November, 2020, and January 2021. During the survey period in Qazvin, COVID-19 caused 1697 deaths, 24 448 hospitalizations, and 136,625 positive test results for COVID-19 infection. Approximately 120,000 older adults currently reside in Qazvin. Using an Integrated Health System (IHS: SIB in Persian: http://10.124.253.30/home/login), 1000 older adults were randomly selected from the IHS records. Using 95% confidence interval with 4% of margin of error and 50% population proportion, the needed sample size for the older population residing in Qazvin was 598. After considering the potential declines, the research team decided to invite 1000 older adults. All information related to households, health houses, and the type of health services required in community health centers are entered and registered in the IHS. Moreover, all household folders related to vital events and received health services are collected in the IHS. To be included in the present study, participants were required to meet the following conditions: aged 65 years or above, can understand and speak Persian, and have the access to short message service and possess smartphone. Healthcare workers sent to older adults a short message service, which contained a direct link to the online survey. Those who agreed to participate directly logged onto a *Google Forms* website to consent and respond. For the 1000 invited participants, 696 agreed to participate in the study and completed the study measures. Therefore, the response rate was 70%. No significant differences were found between the respondents and the non-respondents regarding their age (69.56 vs. 70.01 years) and gender (57.9% vs 59.87 women).

### Measures

Information concerning the demographic variables showing as follows was collected: gender, age, marital status, education, accommodation (rural or urban), occupation, and comorbidity.

The Duke University Religion Index (DUREL)^16^ is five items rated using 5- and 6-point scales. Total scores range between 5 and 27, and a higher score indicates higher religiosity. An example item is *I try hard to carry my religion over into all other dealings in life*. In its Persian language version, was it internally consistent, consistent over time, what was the reliability? What was the indication of validity, such as consistence with other measures? Did this test involve adults, older adults, students, what sample?^31^ The internal consistency of the DUREL in the present sample was 0.84.

The Spiritual Coping Strategy (SCS) scale^32^ is a self-report scale with 20 items rated using a four-point scale (item scores ranging between 0 and 3; nine items on religious coping and another 11 on non-religious coping). A higher score on the SCS indicates a higher level of religious coping (total scores range between 0 and 27) or non-religious coping (total scores range between 0 and 33). An example item is “Maintaining relationship with God and/or higher power, as the source of strength and hope”. Moreover, the psychometric properties of the SCS have been supported among Iranians in its Persian language version after the Persian SCS underwent a rigorous and standard translation procedure including forward translation, back translation, committee panel, cognitive interview, and pilot testing.^33^ The internal consistency of the SCS in the present sample was 0.89.

Fear of COVID-19 was assessed using the Fear of COVID-19 Scale (FCV-19S).^34^ The FCV-19S is a self-report scale with seven items rated using a five-point scale (item scores ranging between 1 and 5). A higher score on the FCV-19S indicates a higher level of fear (total scores range between 7 and 35). An example item is “I am most afraid of coronavirus-19”. Moreover, the FCV-19S was originally developed in Persian among Iranians and the psychometric properties of the original FCV-19S have been supported among Iranians.^34^ The internal consistency of the FCV-19S in the present sample was 0.91.

Depression and anxiety were assessed using the anxiety and depression subscales of the Hospital Anxiety and Depression Scale (HADS).^35^ The HADS is a self-report scale with 14 items rated using a four-point scale (item scores ranging between 0 and 3; seven items for anxiety and seven items for depression). A higher score on the HADS indicates a higher level of anxiety (total scores range between 0 and 21) or depression (total scores range between 0 and 21); a subscale total score higher than 8 further indicates at-risk of anxiety or depression.^36^ An example item is “I feel tense or wound up”. Moreover, the psychometric properties of the HADS have been supported among Iranians in its Persian language version after the Persian HADS underwent a rigorous and standard translation procedure including forward translation, back translation, committee panel, cognitive interview, and pilot testing.^37^ The internal consistency of the HADS in the present sample was 0.85.

### Statistical analysis 

**Figure 1:**
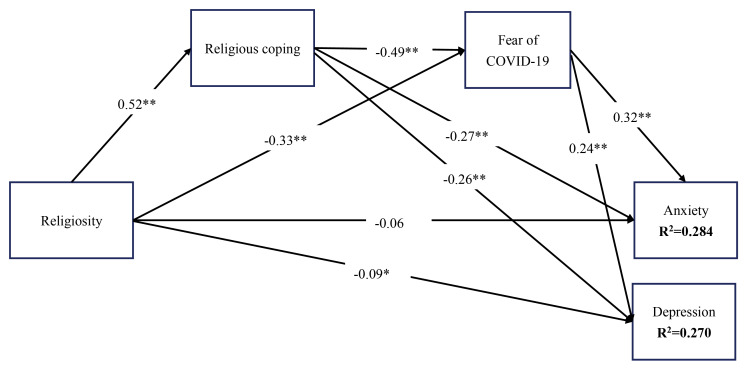
Results of standardized coefficients for the proposed mediation model with spiritual coping and fear of COVID-19 as proposed mediators. Model fit: χ^2^ (df)=3.396 (1); *P* value < 0.001; standardized root-mean-square residual = 0.051; root mean square error of approximation 90% CI = 0.041 (0.039-0.043); Tucker-Lewis index = 0.966; comparative fit index = 0.976. *P< 0.05; **P< 0.001, controlling for gender, marital status, age, accommodation, occupation, comorbidity and educational status

Structural equation modeling^38^ was used to examine the how religious coping and fear of COVID-19 mediated the association between religiosity and anxiety and that between religiosity and depression (see Figure 1 for the entire conceptual model) controlling for age, sex, marital status, accommodation, occupation, comorbidity, and educational status. In the structural equation modeling, parameters were estimated using the full information maximum likelihood (FIML) estimator. The mediated effects were tested using the bootstrapping method was used to determine whether they are significant. More specifically, a total of 10 000 bootstrapping samples with bias corrected confidence interval were generated and if the two limits of 95% confidence interval do not cover 0, the mediated effect is supported.^39^ In addition, fit statistics including standardized root-mean-square residual (SRMR), root mean square error of approximation (RMSEA), comparative fit index (CFI), and Tucker-Lewis index (TLI) were performed. More specifically, a supported model should have CFI and TLI over 0.9 together with RMSEA and SRMR below 0.08. Statistical analyses were conducted using SPSS and Amos v. 24.

## Results

The entire dataset had less than 5% of missing values and the missing information was taken care of by the FIML estimator in the structural equation modeling. Moreover, the studied variables were assumed as normally distributed because of the non-significant results in the Mardia’s multivariate test (value=-1.793; *P*> 0.05). The mean age of the participants was 69.56 years (SD = 9.31) with a relatively balanced ratio between men and women (57.9% women). On average, the participants received 7.12 years of education (SD = 4.03). Moreover, most of the participants were currently married (78.08%), followed by those currently widowed (19.54%). Three-quarters of the participants lived in an urban area (75.14%) and more than three-quarters of the participants were retired (77.30%). Less than 5% of the participants had no comorbidity with most participants reporting one or two comorbidities (85.06%). Moreover, using a cutoff of 8, the prevalence of at-risk anxiety was 28.2% (n = 195); that of at-risk depression was 31.9% (n = 221). The mean average and SD scores on religiosity, anxiety, religious coping, fear of COVID-19, and depression are presented on Table 1.

**Table 1:** Zero-order correlations, partial correlations, and mean (standard deviation) for religiosity, fear of COVID-19, religious coping, anxiety, and depression

	**Religiosity**	**Religious coping**	**Fear of COVID-19**	**Anxiety**	**Depression**
Zero-order correlation					
Religiosity	--	0.517**	-0.382**	-0.358**	-0.362**
Religious coping	--	--	-0.358**	-0.381**	-0.469**
Fear of COVID-19	--	--	--	0.484**	0.461**
Anxiety	--	--	--	--	0.675**
Depression	--	--	--	--	--
Partial correlations					
Religiosity	--	0.483**	-0.361**	-0.360**	0.359**
Religious coping	--	--	-0.354**	-0.380**	-0.460**
Fear of COVID-19	--	--	--	0.482**	0.457**
Anxiety	--	--	--	--	0.671**
Depression	--	--	--	--	--
Mean (SD)	16.99 (7.02)	15.70 (8.16)	15.95 (6.95)	12.82 (7.54)	11.51 (7.08)

** *P*< 0.001.

Table 1 additionally shows the zero-order correlations between every two variables that were used for the mediation analyses. All the correlations were significant and the magnitude ranged between 0.358 and 0.675. Moreover, anxiety and depression were positively correlated with fear of COVID-19 (0.484 and 0.461, respectively) but negatively correlated with religiosity (-0.358 and -0.362, respectively) and religious coping (-0.381 and -0.469, respectively).

After controlling age, sex, marital status, accommodation, occupation, comorbidity, and educational status, the mediation model showed that religiosity had significant total effects on anxiety (B [SE]= -0.360 [0.035]; *P*= 0.002) and depression (B [SE]= -0.365 [0.034]; *P*= 0.002). Additionally, religiosity had no direct effects on anxiety (B [SE]= -0.063 [0.036]; *P*= 0.072) but had direct effects on depression (B [SE]= -0.087 [0.037]; *P*= 0.023). Moreover, both religious coping and fear of COVID-19 were significant mediators in the association between both religiosity and anxiety, and between religiosity and depression (Table 2). Figure 1 further shows the associations using standardized coefficients between every two studied variables in the mediation model. In addition, the mediation model was fully supported by the satisfactory fit indices (SRMR= 0.051; RMSEA= 0.041; TLI= 0.966; CFI= 0.976) and the variance accounted for was relatively strong for both anxiety (R^2^= 0.284) and depression (R^2^= 0.270).

**Table 2:** Model of the associations between religiosity and psychological distress with fear of COVID-19 and religious coping as the mediators

**Variable**	**Coefficient**	**SE**		***P***
**Anxiety**				
Total effect of religiosity	-0.360	0.035		0.002
Direct effect of religiosity	-0.063	0.036		0.072
Indirect effect of religiosity	**Effect**	**Bootstrapping SE**	**Bootstrapping LLCI**	**Bootstrapping ULCI**
Indirect effect via religious coping	-0.150	0.024	-0.190	-0.112
Indirect effect via fear of COVID-19	-0.166	0.028	-0.213	-0.121
Indirect effect via religious coping and fear of COVID-19	-0.297	0.025	-0.338	-0.095
**Depression**	**Coefficient**	**SE**	***t***	***P***
Total effect of religiosity	-0.365	0.034	-10.735	0.002
Direct effect of religiosity	-0.087	0.037	-2.351	0.023
Indirect effect of religiosity	**Effect**	**Bootstrapping SE**	**Bootstrapping LLCI**	**Bootstrapping ULCI**
Indirect effect via religious coping	-0.198	0.022	-0.235	-0.162
Indirect effect via fear of COVID-19	-0.150	0.029	-0.196	-0.100
Indirect effect via religious coping and fear of COVID-19	-0.280	0.027	-0.327	-0.239

ULCI: upper limit confidence interval; LLCI: lower limit confidence interval; SE: standard error Note: All coefficients and effects are unstandardized coefficients/effects. A total of 10 ,000 bootstrapping samples were used. The model was controlled for age, sex, marital status, accommodation, occupation, comorbidity, and educational status.

## Discussion

Guided by the TMT, the present study proposed a mediation model to examine the associations between religiosity, religious coping, fear of COVID-19, and psychological distress of anxiety and depression among older adults during COVID-19 pandemic. The hypotheses based on the psychological model proposed were all supported by the present findings. More specifically, the mediation model showed that religiosity negatively associated with psychological distress via both religious coping and fear of COVID-19. Moreover, the association between religiosity and depression was only supported by an indirect effect from the aforementioned two mediators but not a direct effect. The association between religiosity and anxiety was supported by both direct and indirect effects from religious coping and fear of COVID-19.

Prior evidence has shown that religiosity can facilitate religious coping.^22-24^ The findings of the present study agree with prior evidence and extends this finding to older adults during the COVID-19 pandemic. Because religiosity facilitates individuals to embrace beliefs and creeds, they are likely to find different ways related to religiosity to cope with their difficulties, especially during a hard and tough period,^25^ such as the COVID-19 pandemic. In this regard, when individuals have higher levels of religiosity, they are prone to have more religious coping as compared with those whose religiosity is lower.

The findings further showed that religious coping, in turn, appears to assist older adults in diminishing the psychological distress, which also echoes prior evidence.^19,26^ Through religious coping, individuals are likely to shift their focus from the suffering and difficulties during the tough time to a more meaningful attitude (e.g., viewing it as a training from God).^22-24^ Therefore, they are likely to reduce their psychological distress because they have reset their mindsets into a more positive rather a negative way. Consequently, individuals can maintain or improve their psychological well-being by using religious coping because they feel that the suffering is bearable.^25^

Fear of COVID-19 in the present study was a significant mediator that mediated the association between religiosity and psychological distress. This finding was anticipated. More specifically, there is ample evidence in recent studies showing the importance of fear of COVID-19 in psychological distress.^3,27,28^ Therefore, the older adults in the present study were likely to have elevated psychological distress, which is very likely to be resulted from their fear of COVID-19. In addition, prior evidence shows that individuals can use religiosity and religious coping to cope with the elevated stress.^19,26^ Given that fear of COVID-19 can be viewed as a type of stress, it is reasonable that higher levels of religiosity and religious coping lead to lower levels of fear of COVID-19. Therefore, it is reasonable to assume that fear of COVID-19 is a significant mediator in the association between religiosity, religious coping, and psychological distress.

According to the present study’s findings, some implications can be made as follows. First, healthcare providers can design some programs to teach older adults who have high levels of religiosity how to use religious coping when they have high levels of psychological distress. For example, healthcare providers may guide older adults how to use some behaviors (e.g., praying) to change their focus from suffering to their beliefs. Second, for those older adults with low levels of religiosity, healthcare providers may consider providing them resources (e.g., providing psychological support or sufficient personal protective equipment to reduce fear of COVID-19 among older adults to cope with their fear of COVID-19. Third, healthcare providers may want to design some education programs for older adults to obtain correct knowledge on COVID-19, including the protective COVID-19 infection behaviors. Therefore, older adults may reduce their fear because they are equipped by the correct knowledge concerning COVID-19.

The present study has some limitations that should be disclosed. First, the present study used a cross-sectional design, therefore, the findings of the present study cannot provide any evidence to support any causal relationships. In other words, the mediation models tested in the present study were solely based on theoretical background in terms of the sequence between studied variables. Second, all participants were older Iranians and the generalizability of the present study findings might be hard to extend to other populations. Indeed, given that the severities of the COVID-19 pandemic have been different across countries,^40^ the mediation models tested in the present study should be examined among other populations. Third, all the measures were assessed using self-report questions and scales, and the commonly encountered methods biases from these types of data cannot be controlled for. More specifically, recall bias, common methods bias, and social desirability bias could have an impact on the present study’s findings.

## Conclusion

Religiosity could be a protective factor among older adults in developing good mental health during the COVID-19 pandemic. More specifically, when older adults have higher levels of religiosity, they are less likely to have psychological distress such as anxiety and depression. Moreover, the associations between religiosity and psychological distress were mediated via religious coping and appear to decrease the fear of COVID-19. In this regard, healthcare practitioners may want to assist older adults in obtaining their resources for coping (e.g., providing sufficient personal protective equipment) during the COVID-19 pandemic. Subsequently, older adults can be prevented from developing serious mental health problems during this tough period and the cost of healthcare sources can be saved.

## Acknowledgements

The authors express gratitude to the study participants who took time to complete the questionnaire. Further, we would like to thank Zainab Alimoradi for their hard work and support of the study.

## Funding

The authors received no financial support for the research, authorship, and/or publication of this article.

## Competing interests

The authors declared no potential conflicts of interest with respect to the research, authorship, and/or publication of this article

## Ethical approval

All procedures were carried out in compliance with the Helsinki Declaration. The Qazvin university of medical sciences Ethics Committee approved the study protocol (reference number: IR.QUMS.REC.1399.542). All information of the participants is confidential and cannot be provided for any other commercial or scientific use.

## Authors’ contributions

KAG and AHP conceived and designed the study. MS, CHU and AHP conducted the literature search, and collected and organized data. CYL and AHP assisted in the data analysis, statistical analysis and data interpretation. CHU, CYL and AHP prepared tables and figures. CHU, CYL and AHP assisted in manuscript preparation and devised the initial version of the manuscript. KAG, CHU, MS, CYL, MDG and AHP edited, revised and approved the final draft of the article.
